# Anxiety and Depressive Symptoms in Moroccan Patients Following Major Lower Limb Amputation: A Three-Month Follow-Up

**DOI:** 10.7759/cureus.60284

**Published:** 2024-05-14

**Authors:** Mohammed Elaatmani, Aziz Ahizoune, Amine El Maqrout, Mohamed Kharmaz, Radouane Abouqal, Khalid Abidi

**Affiliations:** 1 Laboratory of Biostatistics, Clinical Research and Epidemiology, Faculty of Medicine and Pharmacy, Mohamed V University, Rabat, MAR; 2 Neurology, Mohamed V Military Teaching Hospital, Mohamed V University, Rabat, MAR; 3 Trauma and Orthopedics, Ibn Sina University Hospital, Rabat, MAR

**Keywords:** symptom, depression, anxiety, lower limb, major amputation

## Abstract

Objective: Major amputation of a lower limb is a traumatic experience that causes physical and psychosocial disabilities. This study set out to ascertain how anxiety and depression symptoms changed during the three months following the amputation.

Materials and methods: A prospective longitudinal observational study was conducted between October 1, 2019, and January 1, 2021, in the Department of Vascular Surgery and the Department of Orthopedic Traumatology of the Ibn Sina Hospital Center in Rabat, Morocco. The study assesses symptoms of anxiety and depression in patients who have undergone a major lower limb amputation over a three-month interval.

Results: In patients who had undergone a major lower limb amputation, the prevalence of anxiety and depression symptoms was very high immediately postoperatively (47.4% and 79.2%, respectively), with a significant decrease in these symptoms. Three months later, anxiety was reported in 24.4% of cases, and depressive symptoms in 65.1% of cases. Age, amputation level, stump pain, phantom limb pain, re-amputation, and emergency amputation were all associated with an increased risk of anxiety and depression. The patient's psychological preparation prior to the amputation, the anesthetic technique used during the procedure, the patient's mobility, and the patient's post-amputation professional status were all protective factors.

Conclusion: Our research findings bolster the necessity of promptly evaluating and managing anxiety and depression in the initial three months following major lower limb amputation. Thus, we believe that amputee patients ought to receive a formal psychological evaluation, which could be helpful, particularly for those whose anxiety or depression symptoms did not improve after three months.

## Introduction

Major lower limb amputation (MLLA) is a surgical procedure that involves removing part of the different structures constituting the limb [1]. MLLA is an amputation above the ankle that creates instability in walking due to the absence of foot talent [2]. More than half of MLLA is secondary to peripheral vascular disease, occurring alone or in association with diabetes mellitus [3,4]. Trauma and tumor pathology are the second cause of amputation [5]. Concerning the effect of the anesthetic technique used during the amputation procedure, studies have not demonstrated any benefit of the locoregional block on mortality, but have reported a positive impact on morbidity, with a reduction in the length of hospitalization, the length of stay in intensive care, and cardiac and pulmonary complications in patients amputated under locoregional anesthesia [6]. MLLA is a traumatic and stressful experience that results in serious problems with physical functioning, employment status, body image [7], psychological reactions [8], phantom pain [5], residual pain [9], and a limitation in daily activities [10-12]. However, physical and psychological reactions to amputation vary greatly from one individual to another. Certain psychological reactions appear in almost all cases. The most typical psychological responses are anxiety, depression [8,13,14], difficulties in social adaptation, and reduced quality of life [4,5].

Anxiety or anxiety disorder is characterized by emotional, physical, and behavioral symptoms that create a feeling of unease, fear, or worry. These symptoms can worsen into an anxiety attack (also called a panic attack). Depression manifests itself as low energy and mood, low self-esteem, and loss of interest or enjoyment in usual activities. Anxiety and depression can be measured by specific scales such as the Hospital Anxiety and Depression Scale (HADS) [15]. Anxiety and depression harm the quality of life of amputees. This is dependent on factors such as age, gender, social support [16], coping ability [17], pain [18], and the cause and level of amputation [11].

Understanding the psychological consequences following an amputation is essential for improving patient care and shaping public health policies in Morocco. Despite the substantial effects of MLLAs on the mental well-being of individuals, there is a noticeable lack of national data regarding the prevalence of depression and anxiety within this population in Morocco. This deficiency in knowledge impedes our capacity to create targeted interventions and support systems that cater to the specific requirements of lower-limb amputees. Through an examination of the progression of anxiety and depression symptoms immediately post-surgery and three months thereafter, our research seeks to illuminate the psychological hurdles encountered by these individuals and pinpoint factors that could impact their mental health. Such insights are crucial for healthcare professionals to devise efficient strategies for early identification, intervention, and sustained management of psychological distress in lower limb amputees.

## Materials and methods

Study design

A prospective single-center and observational study was carried out between October 1, 2019, and January 1, 2021, in the Department of Vascular Surgery and the Department of Orthopedic Traumatology of the Ibn Sina Hospital Center in Rabat, Morocco. Two interviews were carried out, three months apart. The first assessment was conducted within 48 hours after MLLA (T0), face-to-face with the investigator (who has a clinical psychology degree). The second assessment was carried out three months later (T1) during the follow-up consultation or by telephone with the same investigator.

The approval was obtained from the ethics committee for biomedical research of the faculty of medicine and pharmacy of the Mohammed V University of Rabat, Morocco (approval number: 7/20). The ethics committee is registered with the Office for Human Research Protections of the U.S. Department of Health and Human Services under number IORG0006594 (http://ohrp.cit.nih.gov/search/search.aspx).

Inclusion and exclusion criteria

We included patients aged 18 years or older with unilateral or bilateral MLLA, regardless of the cause of amputation. The first assessment during our study period of these latter patients was considered T0. We excluded patients who refused to participate and those with neuropsychiatric disorders. Also, patients undergoing medical treatment may experience heightened levels of anxiety or depression due to certain factors, such as the use of contraceptive hormones, specific high-blood pressure medications like metoprolol and atenolol, cough suppressants such as hydrocodone, and medications for gastroesophageal reflux like omeprazole. Additionally, individuals who encounter postoperative complications necessitating admission to the intensive care unit may also be at risk for psychological distress. Finally, patients who had difficulty understanding Arabic and were not screened or assessed within the first 48 hours were also excluded.

Data collections

The survey consisted of questions on various socio-demographic data such as age, gender, education level, marital status, comorbidities, and tobacco consumption. The clinical data survey included information about the type of amputation, the limb that was amputated, the type of anesthesia used, the level of amputation, the reason for amputation, and the presence of pain in the stump or phantom limb. Moreover, the survey gathered data regarding the patient's post-amputation procedure, such as their ability to use the restroom, dress themselves, and move around (Appendices).

To better understand the symptoms of anxiety and depression in patients who have undergone MLLA, a study investigator conducted an interview using a diagnostic questionnaire for anxiety and depression. The HADS score was used to assess symptoms of anxiety and depression. The HADS score consists of two separate sections that measure the cognitive and emotional aspects of depression (HADS-D) and anxiety (HADS-A). Each section includes a four-point Likert rating scale (0 to 3 points). High scores in these sections indicate a high risk of depression and anxiety [15]. The HADS has been reported to have high internal consistency (Cronbach's alpha; HADS-D: 0.67-0.90, HADS-A: 0.68-0.93) [19]. The HADS scale has been used extensively in studies of people who have undergone amputation [11,20-23]. An Arabic version was utilized to determine the presence of anxiety and depression symptoms, with good internal consistency and reliability (Cronbach's alpha of 0.87 for the anxiety subscale and 0.84 for the depression subscale) [24].

Statistical analysis

Continuous variables were presented as mean, and standard deviation, while dichotomous data were presented as frequency and percentage. The prevalence of anxiety and depression symptoms at T0 and T1 was estimated using the authors' cutoff scores (patients who had a HADS-A and HADS-D score greater than or equal to 11). The HADS-A and HADS-D scores were compared using the Wilcoxon test, the paired sample t-test for the HADS variable, and the Mcnemar test for the stump pain and phantom limb pain variables at T0 and T1. For regression, univariate analysis by the generalized linear model was used to find the factors that influence symptoms of anxiety and depression in T0 and T1. The variables included in the multivariate regression model showed a significant correlation (with a value of p<0.20, which was considered significant) with the dependent variables and had relevance according to the literature. A value of p<0.05 was considered significant.

## Results

At baseline, at T0, 101 patients who underwent MLLA participated in the study. Among them, 64 (63.4%) underwent trans-tibial amputation, 33 (32.6%) trans-femoral, and four (4%) hip disarticulation. Diabetic foot complication was the main cause of amputation, followed by isolated vascular pathology, then tumor pathology and trauma with percentages of 47.5%, 31.7%, 11.9%, and 8.9% respectively. Re-amputation of the lower limb was performed in 84 (83.2%) patients. Eighty-four (83.2%) amputee patients had at least one associated medical condition (Table 1). Among the 101 amputee patients, only 86 (85.15%) participated in the second evaluation. The median length of stay was 11 [6-21] days. The other sociodemographic and clinical variables linked to symptoms of anxiety and depression at T0 and T1 are represented in Table 1. Three-month mortality was 12.9% (13 patients).

**Table 1:** Prevalence of symptoms of anxiety and depression according to sociodemographic and clinical variables after major lower limb amputation at T0 and T1. T0: Immediate post-amputation; T1: Evaluation three months after hospital discharge. Psychological preparation means an explanation of the amputation procedure and/or taking an anxiolytic before lower limb amputation.

Variables	Anxiety		Depression	
	T0 n (%)	T1 n (%)	T0 n (%)	T1 n (%)
Sex	58 (57.4)	21 (24.4)	80 (79.2)	56 (65.1)
-Men	41 (70.7)	18 (85.7)	55 (68.8)	36 (64.3)
-Women	17 (29.3)	3 (14.3)	25 (31.2)	20 (35.7)
School level				
-Illiterate	22 (37.9)	9 (42.9)	43 (53.8)	25 (44.6)
-Primary	19 (32.8)	6 (28.6)	22 (27.5)	14 (25)
-Secondary	12 (20.7)	3 (14.3)	9 (11.3)	11 (19.7)
University	5 (8.6)	3 (14.3)	6 (7.5)	6 (10.7)
Marital status				
-Bachelor	8 (13.8)	4 (19)	7 (8.8)	7 (12.5)
-Bride	35 (60.3)	15 (71.4)	55 (68.8)	40 (71.4)
-Divorcee	5 (8.6)	0	6 (7.5)	2 (3.6)
-Widower	10 (17.2)	2 (9.5)	12 (15)	7 (12.5)
Comorbidity	50 (86.2)	18 (85.7)	62 (81.2)	44 (78.6)
-Diabetes	21 (36.2)	8 (38.1)	28 (35)	21 (37.5)
-Heart disease	27 (65.9)	11 (52.4)	34 (42.5)	17 (30.4)
-Respiratory pathology	6 (10.3)	0	7 (8.8)	4 (7.1)
-Stroke	1 (1.7)	1 (4.8)	3 (3.8)	2 (3.6)
-High blood pressure	29 (50)	11 (52.8)	40 (50)	22 (39.3)
-Dyslipidemia	15 (25.8)	4 (19)	19 (23.8)	10 (17.9)
-Renal failure	11 (18.9)	3 (14.3)	15 (18.8)	9 (16.1)
Smoking	27 (49.1)	8 (38.1)	41 (51.3)	33 (58.9)
Type of surgery				
-Urgent	18 (22.4)	3 (14.3)	24 (30)	18 (32.2)
-Scheduled	45 (77.6)	18 (85.7)	56 (70)	38 (67.8)
Type of anesthesia				
-Locoregional	9 (15.5)	2 (9.6)	11 (13.8)	6 (10.7)
-Spinal anesthesia	12 (20.7)	3 (14.3)	11 (13.8)	12 (21.4)
General anesthesia	37 (63.8)	16 (76.2)	58 (72.4)	38 (58.9)
Amputation level				
-Transtibial	35 (60.4)	13 (61.2)	55 (68.8)	32 (57.1)
-Transfemoral	22 (37.9)	8 (38.1)	25 (31.3)	23 (41.1)
-Hip disarticulation	1 (1.7)	0	0	1 (1.8)
Cause of amputation				
-Vascular diabetes	30 (51.7)	10 (47.6)	38 (47.5)	23 (41.1)
-Vascular	16 (27.6)	8 (38.1)	28 (35)	19 (33.9)
-Traumatic	4 (6.9)	2 (9.6)	8 (10)	7 (12.5)
-Tumoral	8 (13.8)	1 (4.8)	6 (7.5)	7 (12.5)
Complication	31 (53.5)	12 (57.2)	40 (50)	27 (48.2)
Reamputation	48 (82.8)	1 (4.8)	70 (87.5)	48 (85.7)
Psychological preparation				
-Yes	3 (5.2)	2 (9.5)	2 (2.5)	3 (5.4)
-No	55 (94.8)	19 (90.5)	78 (97.5)	53 (94.6)
Stump pain	17 (29.3)	9 (42.9)	38 (47.5)	25 (44.7)
Phantom limb pain	34 (58.6)	19 (90.5)	55 (68.8)	52 (92.9)

The prevalence of anxiety symptoms at T0 and T1 was respectively 47.4% and 24.4% for amputee patients with a HADS-A score that exceeds the threshold of 11. In the immediate postoperative period, the prevalence of anxiety symptoms in amputee patients requiring assistance (partial or total) to use the toilet or move around, was high compared to those who did not require it. On the other hand, this prevalence decreased three months after the amputation procedure (Table 2).

**Table 2:** Incidence of anxiety and depressive symptoms linked to physical functioning following major lower limb amputation at T0 and T1. T0: Immediate post-amputation; T1: Three months after hospital discharge.

Variable	Anxiety n (%)		Depression n (%)	
	T0 58 (57.4)	T1 21 (24.4)	T0 80 (79.2)	T1 56 (65.1)
Toilet use				
-Alone	5 (8.6)	12 (57.2)	6 (7.5)	26 (46.4)
-Partial help	24 (41.4)	7 (33.3)	31 (38.8)	22 (39.3)
-Total help	29 (50)	2 (9.5)	43 (53.7)	8 (14.3)
Dressing				
-Alone	14 (24.1)	15 (71.4)	15 (18.7)	35 (62.5)
-Partial help	16 (27.6)	4 (19.1)	32 (40)	18 (32.1)
-Total help	28 (48.3)	2 (9.5)	33 (41.2)	3 (5.4)
Mobility				
-Alone	3 (5.2)	3 (14.3)	3 (3.7)	12 (21.4)
-Partial help	25 (43.1)	11 (52.4)	32 (40 )	27 (48.2)
-Total help	30 (51.7)	7 (33.3)	45 (56.2)	17 (30.4)

The prevalence of depressive symptoms at T0 and T1 was 79.2% and 65.1%, respectively. The prevalence of depressive symptoms in amputee patients requiring assistance (partial or total) to use the toilet, to dress, or to move around, was high immediately postoperatively and three months after amputation (Table 2).

The variables influencing anxiety levels in patients undergoing MLLA were obtained by analyzing data at three months after amputation (T1) as well as at the first assessment (T0). Interestingly, at both time points, age showed a constant negative association with anxiety scores, suggesting that younger patients generally experienced higher anxiety after amputation (Tables 3, 4). 

**Table 3:** Regression using the generalized linear model of the factors influencing anxiety symptoms immediately after a major lower limb amputation. PNB: Peripheral nerve block; SA: Spinal anesthesia; TT: Transtibial; TF: Transfemoral.

Variable	β	95% CI		t	p
		Lower	Upper		
Ordinate at origin	14.589	9.224	19.955	5.402	< .001
Age	-0.063	-0.116	-0.001	-2.368	0.020
Type of anesthesia					
-PNB	-2.893	-5.494	-0.292	-2.210	0.030
-SA	-2.613	-4.654	-0.571	-2.543	0.013
Amputation level					
-TT	4.701	0.902	8.501	2.458	0.016
-TF	6.044	2.350	9.739	3.250	0.002
Stump pain	1.2939	0.002	2.590	1.983	0.050

**Table 4:** Regression using the generalized linear model of the factors influencing anxiety symptoms three months after a major lower limb amputation.

Variable	β	95% CI		t	p
		Lower	Upper		
Ordinate at origin	8.002	5.242	10.761	5.77	< .001
Age	-0.047	-0.092	-0.001	-2.06	0.042
Mobility					
-Alone	1.268	-0.756	3.293	1.25	0.216
-Partial help	1.974	0.453	3.495	2.58	0.012
Exercise of the profession	1.813	0.036	3.589	2.03	0.046
Hospital stay	0.029	-0.007	0.065	1.61	0.111
Lack of psychological preparation	3.283	0.132	6.433	2.07	0.041

At T0, there was a significant correlation between increasing anxiety and the type of anesthesia, specifically peripheral nerve block. Anxiety levels at T0 were correlated with transtibial and transfemoral amputation levels. Anxiety and stump pain were related at T0. The more stump pain, the higher the level of anxiety there was. Lack of psychological preparation and partial mobility assistance were significantly correlated with higher anxiety at T1, and there was a positive association between anxiety and professional engagement. However, hospital stay did not exhibit any noteworthy associations at T1 (Table 4).

Concerning the factors affecting the level of depression in patients undergoing MLLA at T0 and T1. At T0, there was no significant correlation found between depression and age or male gender (Table 5). Significantly greater levels of depression were observed in those with cardiac disease at T1, indicating the influence of this comorbidity on mental health (Table 6). 

**Table 5:** Regression using the generalized linear model of the factors influencing depressive symptoms immediately after a major lower limb amputation.

Variable	β	95% CI		t	p
		Lower	Upper		
Ordinate at origin	8.583	5.48	11.686	5.493	< .001
Age	0.019	-0.0217	0.059	0.919	0.360
Sex	0.725	-0.655	2.105	1.044	0.299
Urgent surgery	1.686	0.23	3.142	2.299	0.024
Amputation level					
-Transtibial	6.468	3.119	9.819	3.835	< .001
-Transfemoral	5.934	2.427	9.438	3.361	0.001
Stump pain	2.135	0.693	3.577	3.577	0.004
Phantom limb pain	1.693	0.221	3.166	3.166	0.025
Reamputation	1.917	0.184	3.65	2.197	0.031

**Table 6:** Regression using the generalized linear model of the factors influencing depressive symptoms three months after a major lower limb amputation.

Variable	β	95% CI		t	p
		Lower	Upper		
Ordinate at origin	8.14	6.159	10.122	8.18	< .001
Reamputation	1.91	0.559	3.261	2.81	0.006
Heart disease	1.79	0.728	2.84	3.36	0.001
Mobility					
-Total help	2.48	0.983	3.979	3.30	0.001
-Partial help	1.25	0.131	2.370	2.22	0.029
Prosthesis fitting	1.51	-0.386	3.404	1.59	0.117

At T0, urgent surgery was linked to a higher level of depression. Stump pain and phantom pain were correlated with increased depression at T0. Transtibial and transfemoral amputations were significantly associated with higher depression at T0 (Table 5). At T1, there was a significant correlation between total and partial mobility assistance and a high level of depression. Prosthesis fitting, however, didn't show any meaningful correlation with depression (Table 6). The reamputation was correlated with a higher level of depression at T0 and T1.

The results indicate that HADS-A and HADS-D scores are significant predictors of the dependent variable (anxiety or depression) at T0. This relationship persists at T1, although it is slightly attenuated (Table 7).

**Table 7:** Stepwise multiple regression analysis model of variables affecting anxiety and depression after a major lower limb amputation (p<0.05). HADS-A: Hospital Anxiety and Depression Scale-Anxiety; HADS-D: Hospital Anxiety and Depression Scale-Depression; T0: Immediate post-amputation; T1: Evaluation three months after hospital discharge.

model	R	Adjusted R²	F	p
HADS-A at T0	0.592	0.350	4.85	< .001
HADS-A at T1	0.435	0.189	3.08	0.009
HADS-D at T0	0.657	0.431	8.72	< .001
HADS-D at T1	0.518	0.268	5.87	< .001

## Discussion

This research aims to assess the symptoms and causes of anxiety and depression in Moroccan patients who have undergone MLLA (major lower limb amputation) immediately after the surgery and three months after discharge. The study revealed that the occurrence of anxiety and depression symptoms was high in the immediate post-amputation phase, with 58 (57.4%) patients showing anxiety symptoms and 80 (79.2%) showing depression symptoms. However, three months later, there was a decrease in the prevalence of these symptoms, with only 21 (24.4%) patients showing anxiety symptoms and 56 (65.1%) showing depression symptoms. The study has identified several factors that affect the levels of anxiety and depression immediately post-amputation and three months later. The factors that are found to influence the level of anxiety immediately post-amputation are age, peripheral nerve block, level of amputation, and stump pain. Age, mobility limitation, and professional practice were found to be the key factors that impacted the level of anxiety three months later. Regarding the factors affecting depression immediately post-amputation, the study found that emergency amputation, stump pain, phantom limb, and re-amputation impact the level of depression. However, three months after discharge, heart disease, re-amputation, and mobility limitation were the factors that influenced the level of depression.

During the early period after surgery, the occurrence of anxiety symptoms in our sample was 58 (57.4%) patients. There has been no study conducted in high-income countries to evaluate anxiety symptoms during the immediate postoperative period of MLLA. However, a similar result was found by Migaou et al. who assessed the symptoms of anxiety at the first consultation, not immediately after amputation, which reported a prevalence of 46.6% [4]. This outcome may be related not only to the patient's tendency to develop anxiety but also to the fear associated with losing a limb, the stress linked to the act of amputation, the experience of being hospitalized, and the fear of changing body image. After three months of undergoing MLLA, the prevalence of anxiety symptoms decreased from 58 (57.4%) to 21 (24.4%). The study also showed a moderate decrease in the prevalence of depression symptoms from 80 (79.2%) cases to 56 (65.1%) cases between T0 and T1. Due to differences in sample size, nature, and time elapsed after amputation, it is not appropriate to compare this study to others. However, a Brazilian study reported a prevalence of around 68.5% [24]. In general, the prevalence of symptoms of anxiety and depression after MLLA gradually decreases, whether at three months [18], six months [21], or one year and more [11,14,21,25-27]. This reduction can be explained by the relief of pain, and the absence of fear and worry after the MLLA. In addition to the negative effects of prolonged hospitalization, which can lead to functional decline and worsening emotional trauma [22].

Concerning the sociodemographic aspect of patients who developed anxiety and depression after MLLA. Our study showed that the majority of patients were men (70.7% at T0 and 85.7% at T1 for anxiety; 68.8% at T0 and 64.3% at T1 for depression) (Table 1). The majority of patients, 83 (82.2%) cases were over 50 years old. These results are consistent with data from the literature [4,13,18,21]. Thus, the level of education is linked to symptoms of anxiety and depression. Indeed, the higher the level of education, the more the symptoms of anxiety and depression are reduced. This result corroborates that demonstrated in the study carried out by Al-Ayed et al. jointly between Saudi Arabia and Jordan [22]. Regarding the cause of amputation, our study showed that patients with vascular and tumor amputations had higher levels of depression and anxiety immediately after MLLA. Also, this high prevalence was observed at three months in amputee patients with vascular etiology. The study conducted by Hawamdeh et al. revealed that the high risk of anxiety and depression was related to the traumatic cause [26]. Our study showed that smoking was responsible for high levels of anxiety and depression. This result was similar to that demonstrated in the study by Al-Ayed et al. [22].

The present study found that age, level of amputation, and stump pain are predictive factors for anxiety levels during immediate MLLA. To our knowledge, no study has been conducted in this direction before. As for the impact of the anesthetic technique used during MLLA, the study revealed that peripheral nerve block and spinal anesthesia are negatively associated with anxiety symptoms at T0. This is perhaps linked to the patient's experience during amputation and postoperative pain. To our knowledge, no study has addressed the effect of anesthetic techniques on the psychological reactions of patients who have undergone MLLA. Factors that negatively impact the level of depression after an MLLA at T0 include stump pain, phantom limb pain, as well as the level of amputation and re-amputation. In this sense, a systematic review revealed that the presence of pain after MLLA influences psychosocial adjustments and social well-being [28]. However, MLLA carried out in an emergency is linked to the level of anxiety, so as the frequency of patients amputated in an emergency increases, the level of anxiety increases.

Regarding the impact of the amputation level on anxiety and depression, the study showed that amputation at the trans-femoral level presents a greater risk of having anxiety symptoms and depression than at the transtibial level. This result agrees with the study of Tutak et al. [11]. In this sense, an Iranian study carried out on veterans who had undergone a unilateral transfemoral amputation showed that depression (32% confirmed depressed and 26% suspected) and anxiety (31% confirmed and 28% suspected) have a high prevalence among participants [29].

Three months post-MLLA, age, and mobility limitations were associated with increased anxiety levels, while heart disease, re-amputation, and limited mobility were correlated with higher depression levels. The study by Singh et al. showed a strong association between depression and the presence of medical illnesses associated with amputation [27]. In the same sense, an Iranian study reported that cardiovascular disease is the most common among patients who develop depression [8]. As for physical activity, the study showed that mobility limitations are linked to a high level of anxiety and depression three months after MLLA. This result corroborates with that found by Migaou et al. [4]. Whereas, exercise of the profession has a positive effect on anxiety symptoms. This result is supported by Srivastava et al., who reported that amputees had more anxiety symptoms if they were unemployed or had low income [18]. The use of a prosthesis, although it is linked to anxiety and/or depression in other studies [28, 30], is not significant in our study because the majority of amputees did not benefit from a prosthesis for several reasons, namely the COVID-19 crisis, lack of financial means, infection, and bad stump.

Limits of study

First, although this is a prospective longitudinal study of amputation, the sample size may be insufficient to detect some relationships. A second limitation of the study is the absence of a comparison group. However, it isn't easy to obtain a suitable comparison group. Studying the relationship between MLLA and psychological dysfunction in this group compared to a comparison group would require the latter to be matched for multiple comorbidities associated with amputation. Indeed, a healthy control group would not necessarily be an appropriate comparison. The third limitation of this study is linked to the lack of awareness of the presence or absence of symptoms of anxiety and/or depression before amputation. A fourth limitation is that improvements in anxiety and depression symptoms in the three-month interval do not necessarily reflect the natural recovery process alone but also unmeasured biopsychosocial interventions implemented in the pre- and postoperative period (medications, advice). However, the three-month assessment appears to be of clinical importance because this is a time interval during which recovery occurs and most patients are actively engaged in the rehabilitation process. Despite all limitations, this study includes participants with various causes of amputation at different ages, which is typical of the variability in many clinical settings. In addition to assessing symptoms of anxiety and depression immediately post-amputation, this has not been studied before.

## Conclusions

The level of anxiety and depression after a major lower limb amputation (MLLA) depends on several factors. This study has identified some of these factors, including age, heart disease, level of amputation, anesthetic technique, pain from the stump and/or phantom limb, re-amputation, and limitation of movement. These factors could promote anxiety and/or depression after amputation. On the other hand, practicing a profession and traveling independently are considered protective factors against anxiety and depression after MLLA. The results of the study emphasize the importance of early evaluation and intervention for anxiety and depression in the first three months after MLLA. Therefore, amputee patients must receive comprehensive care, particularly those whose symptoms of anxiety or depression do not improve after three months.

While the effect of anesthesia on psychological reactions after MLLA has not been studied in the amputee population, it is suggested that conducting a multicenter prospective study with a larger sample size could help to better understand the variations in anxiety and depression symptoms in lower limb amputees over time, and evaluate the determining factors.

## Appendices

Record No:

Anxiety and Depression Symptoms Assessment Questionnaire of Lower Limb Amputees

Note: This questionnaire aims to assess the quality of life of lower limb amputee patients. We would like to remind you that all the information you provide here is protected by strict anonymity and perfect confidentiality. We appreciate your cooperation in completing this questionnaire.

Patient Information:

- Age: ……………… years

- Gender: Male □ Female □

- Education level: Illiterate □ Primary □ Secondary □ University □

- Profession: …………………………

- Marital status: Single □ Married □ Widowed □ Divorced □

- Number of children: ……………

- Religion: Muslim □ Jewish □ Christian □ Atheist □

- Residence: Urban □ Rural □

- Weight: …………kg

- Height: …………cm

- BMI: ……...m/kg²

- Comorbidities:

  - Diabetes: Yes □ Equilibrated □ Non-equilibrated □ Neuropathy of lower limbs associated □

  - Heart disease: Yes □ No □

  - Respiratory pathology: Yes □ No □

  - Stroke (CVA): Yes □ No □

  - Kidney failure: Yes □ No □

  - Smoking: Yes □ No □

  - Obesity (BMI > 30): Yes □ No □

  - Cognitive disorders: Yes □ No □

Surgery Information:

- Type of surgery: Urgent □ Scheduled □

- Amputated limb: Right □ Left □

- Type of anesthesia: General □ Spinal □ Regional □

- Level of amputation: Trans-femoral □ Trans-tibial □ Hip disarticulation □ Knee disarticulation □

- Cause of amputation: Vascular □ Traumatic □ Tumoral □ Infection □ Diabetes □

- Hospitalization day: ………/…………/………………

- Date of amputation: ………/…………/ ………………….

- Date of hospital discharge: …………days.

- Possible complications: Infection □ Reamputation □ Bleeding □

                           Other: …………………………………......

Psychological State Information:

- Were you informed about the surgical procedure? Yes □ No □

  If scheduled surgery, did you receive preparation?

    • Medication: Anxiolytic □ Antidepressant □

                         Other (specify): …………………….........

    • Psychological: Yes □ No □

      If yes, by whom: Medical personnel □ Nursing staff □

                          Other (specify): ……………………………

- Did you have the opportunity to ask for explanations from the personnel about what would happen to your body (amputation)?

  Yes □ No □

  If yes, did this assistance relieve you? Yes □ No □

- How did you feel after the lower limb amputation?

  Very bad □ A little □ Well □ Very well □

Post-Intervention Immediate Evaluations:

- Stump pain: Yes □ No □

- Phantom limb pain: Yes □ No □

- Stress evaluation: Absent □ Low □ Medium □ High □

- Autonomy evaluation:

  Toilet: Alone □ Partial assistance □ Total assistance □

  Dressing: Alone □ Partial assistance □ Total assistance □

  Mobility: Alone □ Partial assistance □ Total assistance □

T0: Evaluation of anxiety and depression in patients with lower limb amputations

Don’t take too long over your replies: your immediate is best.

**Table 8:** First assessment by hospital anxiety and depression.

D	A			D	A	
		I feel tense or 'wound up':				I feel as if I am slowed down:
	3	Most of the time		3		Nearly all the time
	2	A lot of the time		2		Very often
	1	From time to time, occasionally		1		Sometimes
	0	Not at all		0		Not at all
		I still enjoy the things I used to enjoy:				I get a sort of frightened feeling like 'butterflies' in the stomach:
0		Definitely as much			0	Not at all
1		Not quite so much			1	Occasionally
2		Only a little			2	Quite Often
3		Hardly at all			3	Very Often
		I get a sort of frightened feeling as if something awful is about to happen:				I have lost interest in my appearance:
	3	Very definitely and quite badly		3		Definitely
	2	Yes, but not too badly		2		I don't take as much care as I should
	1	A little, but it doesn't worry me		1		I may not take quite as much care
	0	Not at all		0		I take just as much care as ever
		I can laugh and see the funny side of things:				I feel restless as I have to be on the move:
0		As much as I always could			3	Very much indeed
1		Not quite so much now			2	Quite a lot
2		Definitely not so much now			1	Not very much
3		Not at all			0	Not at all
		Worrying thoughts go through my mind:				I look forward with enjoyment to things:
	3	A great deal of the time		0		As much as I ever did
	2	A lot of the time		1		Rather less than I used to
	1	From time to time, but not too often		2		Definitely less than I used to
	0	Only occasionally		3		Hardly at all
		I feel cheerful:				I get sudden feelings of panic:
3		Not at all			3	Very often indeed
2		Not often			2	Quite often
1		Sometimes			1	Not very often
0		Most of the time			0	Not at all
		I can sit at ease and feel relaxed:				I can enjoy a good book or radio or TV program:
	0	Definitely		0		Often
	1	Usually		1		Sometimes
	2	Not Often		2		Not often
	3	Not at all		3		Very seldom

Scoring:

Total score: Depression (D): .............................. Anxiety (A): ................................                                      

0-7     = Normal

8-10   = Borderline abnormal (borderline case)

11-21  = Abnormal (case)

T1: Evaluation after three months

- Stump pain: Yes □ No □

- Phantom limb pain: Yes □ No □

- Stress evaluation: Absent □ Low □ Medium □ High □

- Autonomy evaluation:

  Toilet: Alone □ Partial assistance □ Total assistance □

  Dressing: Alone □ Partial assistance □ Total assistance □

  Mobility: Alone □ Partial assistance □ Total assistance □

  Possible home aids: Yes □ No □

  Return to professional activity: Yes □ No □

- Prosthetic fitting: Yes □ No □

   If yes:

   - Date of decision for prosthetic fitting: ……/………/….................

   - Date of definitive prosthetic fitting: ………/………/……………..

   - Prosthesis selection by: Patient □ Technical team □

   - Donning the prosthesis: Alone □ Partial assistance/Supervision □ Complete assistance □

   - Daily duration of prosthesis wear = ……. Hours.

   - Outcome: Return home □ Death □ (Date: …………………)

   In case of discontinuation of prosthetic fitting, please specify the cause and the time after the beginning of the prosthetic fitting attempt:……………………………………………………………………………………………………………………………………………………………………………………

**Table 9:** Second assessment by hospital anxiety and depression.

D	A			D	A	
		I feel tense or 'wound up':				I feel as if I am slowed down:
	3	Most of the time		3		Nearly all the time
	2	A lot of the time		2		Very often
	1	From time to time, occasionally		1		Sometimes
	0	Not at all		0		Not at all
		I still enjoy the things I used to enjoy:				I get a sort of frightened feeling like 'butterflies' in the stomach:
0		Definitely as much			0	Not at all
1		Not quite so much			1	Occasionally
2		Only a little			2	Quite often
3		Hardly at all			3	Very often
		I get a sort of frightened feeling as if something awful is about to happen:				I have lost interest in my appearance:
	3	Very definitely and quite badly		3		Definitely
	2	Yes, but not too badly		2		I don't take as much care as I should
	1	A little, but it doesn't worry me		1		I may not take quite as much care
	0	Not at all		0		I take just as much care as ever
		I can laugh and see the funny side of things:				I feel restless as I have to be on the move:
0		As much as I always could			3	Very much indeed
1		Not quite so much now			2	Quite a lot
2		Definitely not so much now			1	Not very much
3		Not at all			0	Not at all
		Worrying thoughts go through my mind:				I look forward with enjoyment to things:
	3	A great deal of the time		0		As much as I ever did
	2	A lot of the time		1		Rather less than I used to
	1	From time to time, but not too often		2		Definitely less than I used to
	0	Only occasionally		3		Hardly at all
		I feel cheerful:				I get sudden feelings of panic:
3		Not at all			3	Very often indeed
2		Not often			2	Quite often
1		Sometimes			1	Not very often
0		Most of the time			0	Not at all
		I can sit at ease and feel relaxed:				I can enjoy a good book or radio or TV program:
	0	Definitely		0		Often
	1	Usually		1		Sometimes
	2	Not often		2		Not often
	3	Not at all		3		Very seldom

Scoring:

Total score: Depression (D): .............................. Anxiety (A): ................................                                                       0-7     = Normal

8-10   = Borderline abnormal (borderline case)

11-21 = Abnormal (case)
